# Impact of Elevated CO_2_ and Temperature on Brown Planthopper Population in Rice Ecosystem

**DOI:** 10.1007/s40011-016-0727-x

**Published:** 2016-04-19

**Authors:** G. Guru Pirasanna Pandi, Subhash Chander, Madan Pal Singh, Himanshu Pathak

**Affiliations:** 1 Division of Entomology, Indian Agricultural Research Institute, Pusa Campus, New Delhi, 110012 India; 2 Division of Plant Physiology, Indian Agricultural Research Institute, Pusa Campus, New Delhi, 110012 India; 3 Centre for Environment Science and Climate Resilient Agriculture, Indian Agricultural Research Institute, Pusa Campus, New Delhi, 110012 India

**Keywords:** Basmati rice, Brown planthopper, Climate change, Elevated CO_2_, High temperature

## Abstract

Influence of elevated CO_2_ (570 ± 25 ppm) and elevated temperature (≃3 °C higher than ambient) on rice (*Oryza* *sativa* L.) and brown planthopper (BPH), *Nilaparvata lugens* (Stal.) was studied in open top chambers during rainy season of 2013. Elevated CO_2_ and temperature exhibited positive effect on BPH multiplication thus enhancing its population (55.2 ± 5.7 hoppers/hill) in comparison to ambient CO_2_ and temperature (25.5 ± 2.1 hoppers/hill). Elevated CO_2_ + temperature significantly reduced the adult longevity and nymphal duration by 17.4 and 18.5 % respectively, however elevated conditions increased BPH fecundity by 29.5 %. In rice crop, interactive effect of elevated CO_2_ and temperature led to an increase in the number of tillers (20.1 %) and canopy circumference (30.4 %), but resulted in a decrease of reproductive tillers (10.8 %), seeds/panicle (10.9 %) and 1000-seed weight (8.6 %) thereby reducing grain yield (9.8 %). Moreover, positive effect of increased CO_2_ concentration and temperature on BPH population exacerbates the damage (30.6) which in turn coupled with the plant traits to hampering production.

## Introduction

Rice (*Oryza* *sativa* L.) is indisputably the world’s most important staple food that provides nutrition to more than half of the world’s population [1, 2]. In India, rice is grown on an area of 43.94 million ha with a production of 106.65 million tonnes [3]. Area under scented rice varieties especially Basmati, also known as queen of rice is increasing day by day with the demand from world market along with domestic consumption. On the other hand, rice productivity in India is decreasing due to various abiotic and biotic constraints [4], yet the need for grain will continue to grow in the coming decades, due to the population explosion. In the rice ecosystem, outbreak of brown planthopper, *Nilaparvata lugens* (Stal.) has been observed in recent years especially in North India leading to crop failure [5]. At high population densities, this pest causes hopper burn which may inflict as high as 70 % yield loss [6]. Besides it also transmits viruses such as rice ragged stunt virus (RRSV) and rice grassy stunt virus (RGSV).

According to projections, atmospheric CO_2_ is expected to increase up to 550 ppm by 2050 due to increase in anthropogenic emissions of greenhouse gases, which would also increase the global temperature between 1.8 and 4 °C by the end of the current century [7]. Increase in atmospheric CO_2_ will have a significant impact on C_3_ plants such as rice due to changes in photosynthetic carbon assimilation pattern that leads to increase in biomass and productivity [8, 9], while temperature rise will have an adverse effect on C_3_ plants [10–13]. However, actual effect on plant growth and yield would depend on interaction between CO_2_ and temperature.

In the context of climate change on insects, temperature directly affects them, while CO_2_ affects them through host plants [14]. Temperature is probably the single most inevitable environmental factor that influences insect behaviour, distribution, development, survival, and reproduction [15]. Changes in atmospheric CO_2_ affect not only the plant quality but also the herbivore performance. The Carbon:Nitrogen (C:N) ratio of the plant foliage generally increases when plants are grown in elevated CO_2_ than in ambient. Therefore, rise of CO_2_ and temperature may directly affect the food grain production and indirectly through its effect on crop pests [16]. Earlier reports also suggest that rice production is under severe threat due to anticipated environmental changes [17].

Most researches have focused on the individual effects of CO_2_ and temperature on the crop yield and phenological parameters of plants grown in controlled environments. Works on interactive effects of elevated CO_2_ and temperatures are rare. Besides in Indian context, relatively little work has been done on the impact of climate change on crop-pest interaction. In this perspective, it is imperative to assess the impact of rising atmospheric CO_2_ and temperature on rice and its important sucking pest brown planthopper (BPH).

## Material and Methods

The impact of elevated CO_2_ (570 ± 25 ppm) + temperature (≃3 °C) on brown planthopper population *vis*-*a*-*vis* ambient CO_2_ (397 ± 25 ppm) + ambient temperature was undertaken on rice (*O. sativa* L; variety Pusa 1401) in open top chambers (OTCs) during rainy season (June–October) 2013 at Indian Agricultural Research Institute, New Delhi (28°38′N latitude, 77°09′E longitude, 228.61 m altitude).

Experiment comprised of two OTCs each under elevated and ambient conditions. Out of four OTCs used in the study, two had elevated condition (CO_2_ + temperature) from 10 days after paddy transplanting to harvest, while in other two ambient conditions were maintained. Under each of the conditions, one OTC had BPH infestation, while in other uninfested crop was grown. Paddy nursery was raised in wet nursery beds, as per recommended package of practices [18]. Two twenty two day’s old seedlings were transplanted in plastic pots (22.5 × 15 cm) at 5–6 cm depth and gap filling was done after a week to ensure uniform plant population and pots were irrigated regularly. Ten days after transplanting (DAT) 10 pots were transferred to each of the OTCs under elevated and ambient conditions. Each OTC represented one treatment with 10 pots in each OTCs constituted one replication. Nitrogen (N), phosphorus (P_2_O_5_) and potash (K_2_O) were applied at the recommended dose of 120:60:40 kg/ha. Crop was harvested after maturity in the 2nd week of November and threshing was done manually.

**Fig. 1:**
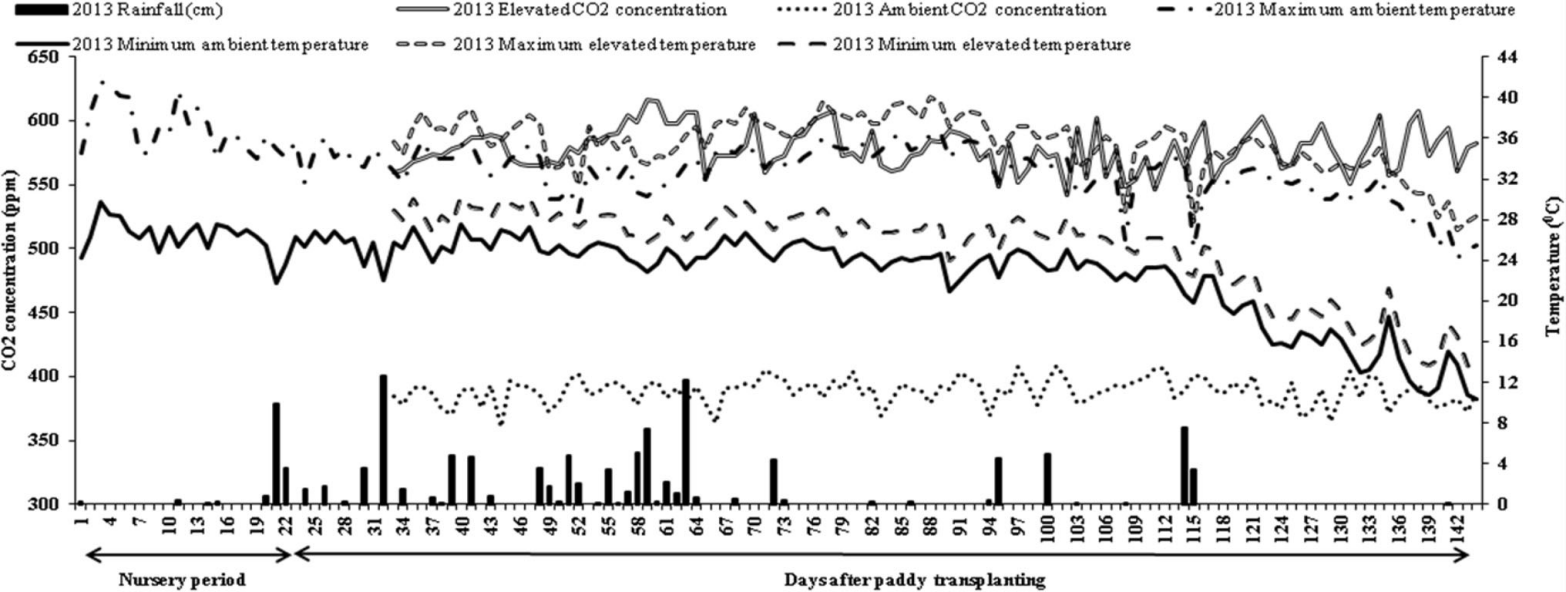
Average daily weather conditions within the OTCs during the study periods

The OTC structure and other details such as CO_2_ supply and monitoring has been described [19, 20]. Upper part of OTCs had a frustum of 0.5 m at 2.5 m height, to reduce the dilution of CO_2_ by air current inside the chambers and was kept open to maintain the near-natural conditions of temperature and relative humidity in ambient OTCs. On the other hand in elevated OTCs, upper two-third portion was covered with polyvinyl chloride (PVC) sheets (120 µm thickness) that transmitted 90 % of natural sunlight to raise the temperature approximately 3 °C more than the ambient. Daily temperature (maximum and minimum) in the OTCs were recorded during the study period (Fig. 1) with the help of sensors (Model TRH 511, Ambetronics, Switzerland) fitted in the middle of each OTC and data logger (Model TC 800D, Ambetronics, Switzerland). To obtain BPH infestation laboratory reared five pairs of BPH adults were released under elevated and ambient condition after 10 days of transfer of pots to OTCs [21]. Weekly observations on number of nymphs, wingless females and males were recorded.

In order to assess the BPH fecundity, one pair of freshly emerged BPH adult was collected from elevated and ambient OTCs and then released on 30-day old rice seedlings in pots under both the conditions in 10 replicates each [22]. Eggs were counted by dissecting scars on leaf sheaths under microscope. Nymphal duration was studied by releasing of ten newly hatched nymphs in each of the 10 experimental pots (13 × 9 cm) under elevated and ambient condition. The pots were covered with mylar cage and observations were taken until adults emergence. Female longevity was studied by maintaining the newly emerged females in their respective pots until they died. The BPH sucking rate was assessed by estimating the amount of honeydew excreted by the adult hoppers. As per the standard protocol [23, 24] five freshly emerged and 3 h pre-starved brachypterous females were allowed to feed for 24 h at the base of the stem. The area of blue rimmed spots that appeared on filter paper as a result of honeydew excretion was measured graphically. The sucking rate was determined by comparing the average area of honeydew excreted in mm^2^.

Observations on plant parameters viz., number of tillers, reproductive tillers, circumference of the hill, canopy circumference, seeds/panicle, 1000-seed weight and grain yield were recorded for each of the 10 plants in the four OTCs. Yield of uninfested and infested plants under elevated as well as ambient conditions were compared to ascertain the effect of elevated condition on the extent of yield loss due to BPH.

### Statistical Analysis

Statistical analyses were performed using SAS Software, Version 9.2 [25]. Repeated measure ANOVA was carried out to assess the interactive effect of elevated condition (570 ± 25 ppm CO_2_ + ≃3 °C) on BPH population, while the effect of CO_2_ and temperature on plant parameters was analysed through t-test [21].

## Results and Discussion

Elevated CO_2_ and temperature exhibited a significant positive effect on BPH multiplication and its population (F = 53.8, LSD = 0.5, *P* < 0.0001) under elevated condition (55.2 ± 5.7 hoppers/hill) than ambient (25.5 ± 2.1 hoppers/hill) throughout the season (Table 1). During the first two weeks, total population of BPH under both conditions did not differ significantly; however, higher BPH population was recorded from third to seventh week after adult release under elevated conditions (F = 9.56, LSD = 1.3, *P* < 0.0001). The BPH was observed to complete two generation during the study period.

**Table 1:** Brown planthopper (BPH) population/hill (nymphs, males and females) in open-top chambers during rainy season 2013

Treatment	BPH Population* (nymphs + males + females)							
	Weeks after adult release							
	1	2	3	4	5	6	7	Mean ± SE
Elevated condition (570 ± 25 ppm + > 3 °C)	2.5 ± 0.6	10.9 ± 1.7	75.8 ± 10.4	169.6 ± 25.3	80.5 ± 13.6	34.4 ± 4.7	12.4 ± 1.1	55.2 ± 5.7
	(1.8 ± 0.2)^h^	(3.3 ± 0.3)^fg^	(8.4 ± 0.6)^b^	(12.5 ± 1)^a^	(8.6 ± 0.7)^b^	(5.7 ± 0.4)^de^	(3.6 ± 0.2)^f^	(6.3 ± 0.3)^a^
Ambient condition (397 ± 25 ppm + > 3 °C)	2.3 ± 0.6	7.7 ± 2.1	31.8 ± 8.5	45.4 ± 7.8	58.5 ± 5.8	28.0 ± 3.9	4.6 ± 1.1	25.5 ± 2.1
	(1.7 ± 0.2)^h^	(2.6 ± 0.3)^fgh^	(5.1 ± 0.7)^e^	(6.6 ± 0.5)^cd^	(7.6 ± 0.4)^bc^	(5.2 ± 0.4)^e^	(2.2 ± 0.2)^gh^	(4.4 ± 0.1)^b^
Mean ± SE	2.4 ± 0.5	9.3 ± 1.5	53.8 ± 7.6	107.5 ± 14.7	69.5 ± 8.2	31.2 ± 2.9	8.5 ± 0.9	
	(1.7 ± 0.2)^f^	(3.0 ± 0.2)^e^	(6.8 ± 0.5)^c^	(9.5 ± 0.6)^a^	(8.1 ± 0.5)^b^	(5.5 ± 0.2)^d^	(2.9 ± 0.2)^e^	

Treatment, F = (53.8), LSD = (0.5), *P* < 0.0001

Week, F = (76.8), LSD = (0.9), *P* < 0.0001

Interaction (treatment × week), F = (9.56), LSD = (1.3), *P* < 0.0001

Planthopper counts with same superscripts do not differ significantly

Data in parenthesis are SQRT (*X* + 1) transformed values

* Mean of ten replications

**Fig. 2:**
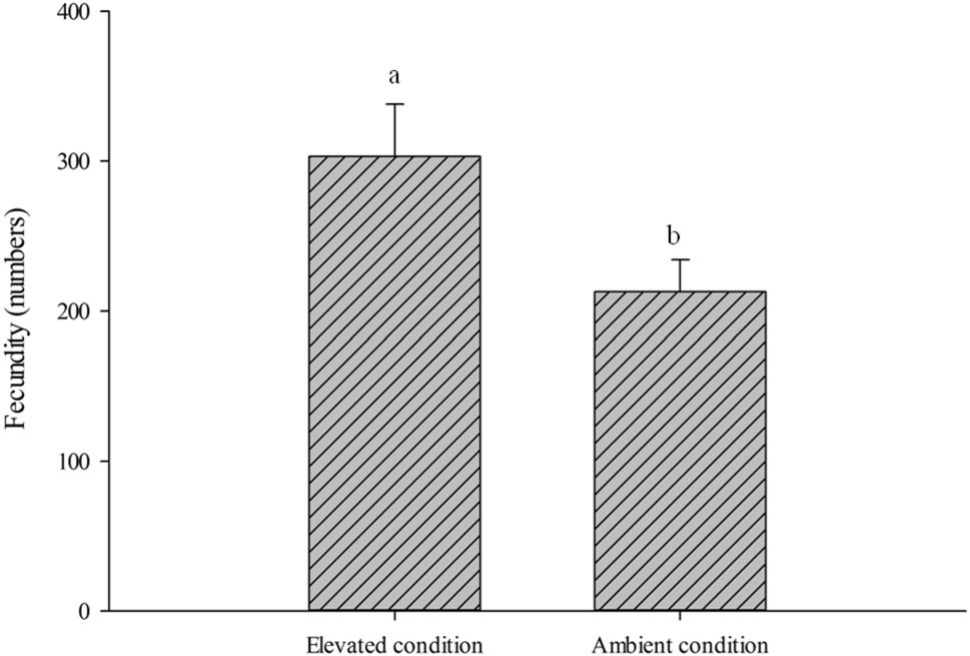
Effect of elevated CO_2_ and temperature on brown planthopper fecundity (mean ± SE)

**Fig. 3:**
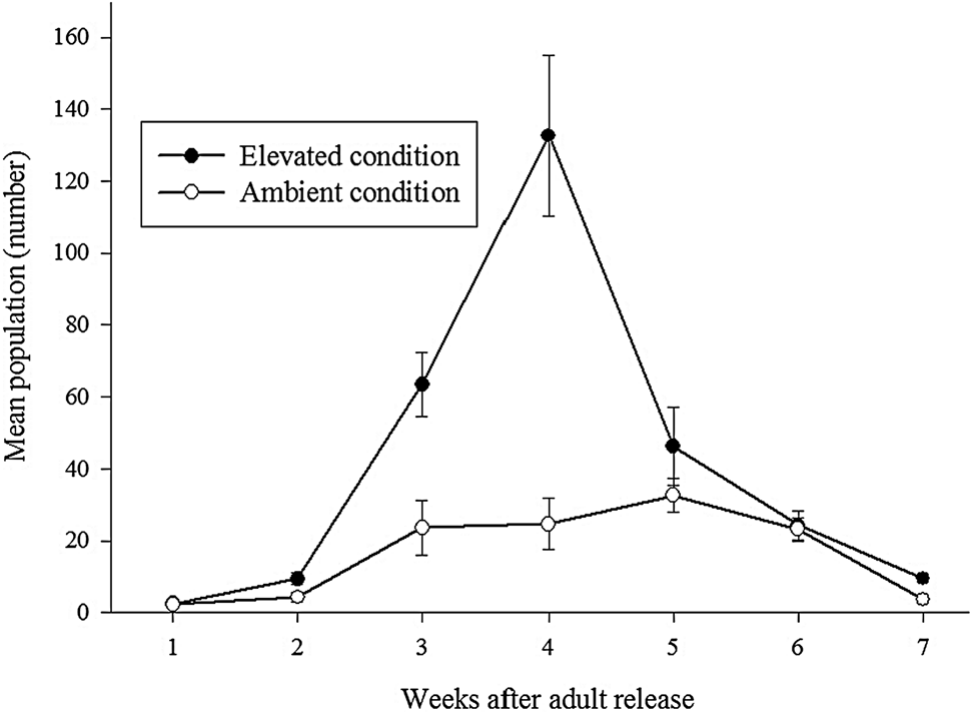
Effect of elevated CO_2_ and temperature on population of the brown planthopper (BPH) nymphal/hill (mean ± SE)

**Fig. 4:**
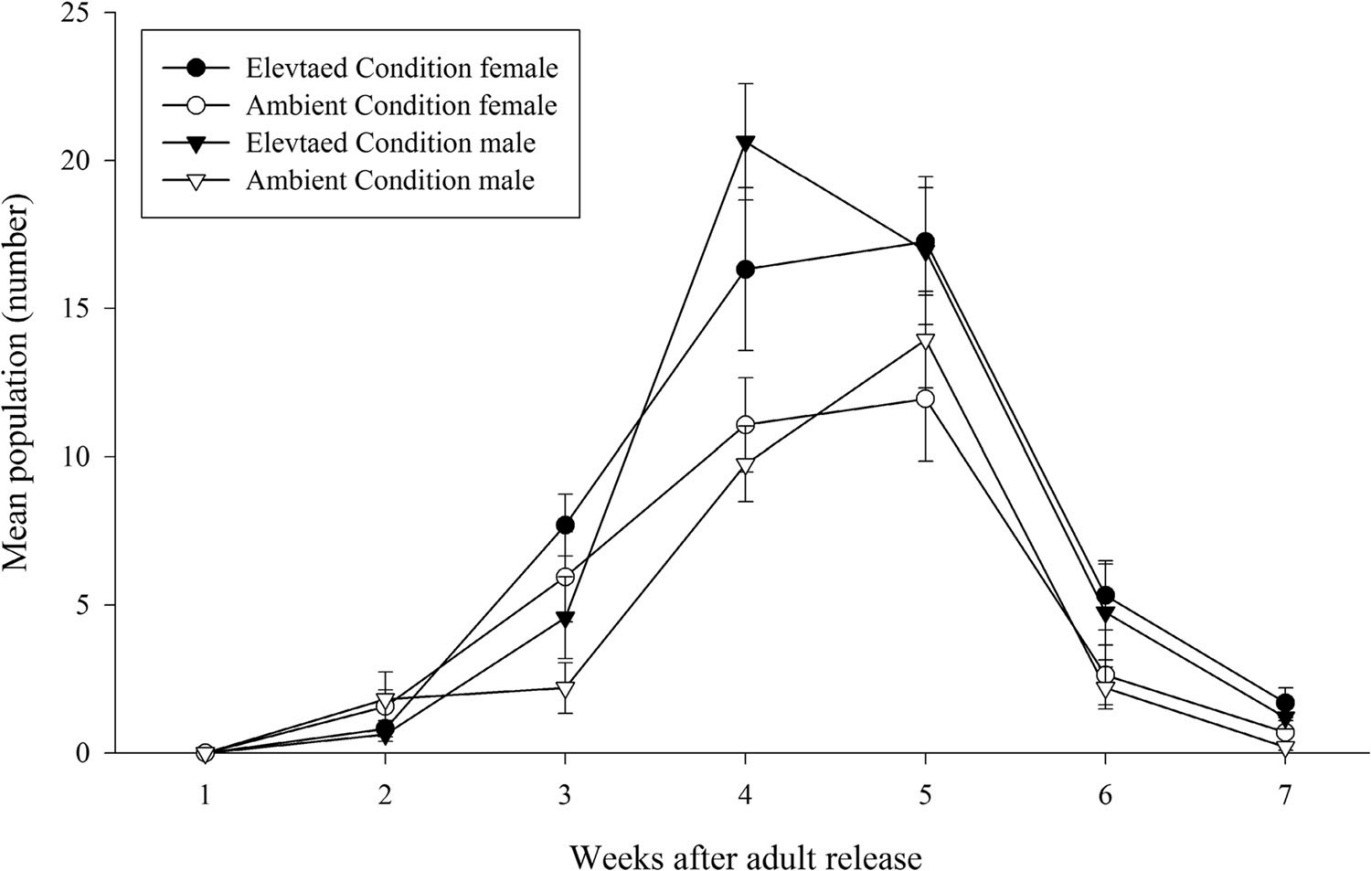
Effect of elevated CO_2_ and temperature on population of the brown planthopper (BPH) male and female/hill (mean ± SE)

The fecundity of brachypterous female differed significantly on the rice plants grown under elevated condition than that of ambient. Rice plants exposed to elevated conditions recorded higher number of eggs (303.2 ± 35 eggs/female) whereas in the plants under ambient condition (212.9 ± 21.5 eggs/female) female laid significantly less number of eggs (t = 2.2, *P* < 0.05). It was revealed that elevated condition stimulated fecundity of BPH by 29.5 % compared to ambient (Fig. 2). Likewise, nymphal population was significantly higher under elevated condition (F = 38.3; *P* < 0.0001) than ambient. Higher fecundity resulted in more nymphal population under elevated condition. Across the weeks, under both conditions higher nymphal population was recorded during fourth week (F = 26.6; *P* < 0.0001) (Fig. 3). Male and female numbers also showed similar trend as to nymphal population build-up. Both male (F = 17.5; *P* < 0.0001) and female (F = 10.1; *P* < 0.001) populations significantly differed under elevated condition than ambient. Across the weeks, higher male (F = 64.4; *P* < 0.0001) and female (F = 44.6; *P* < 0.0001) populations were recorded during fourth and fifth week respectively under both conditions (Fig. 4).

**Fig. 5:**
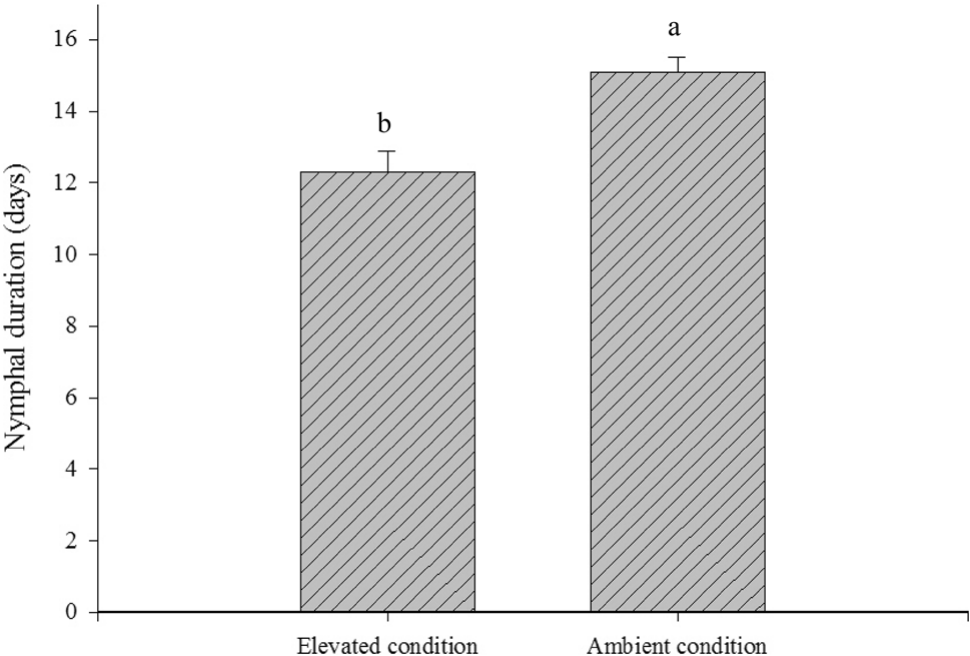
Effect of elevated CO_2_ and temperature on brown planthopper nymphal development (mean ± SE)

**Fig. 6:**
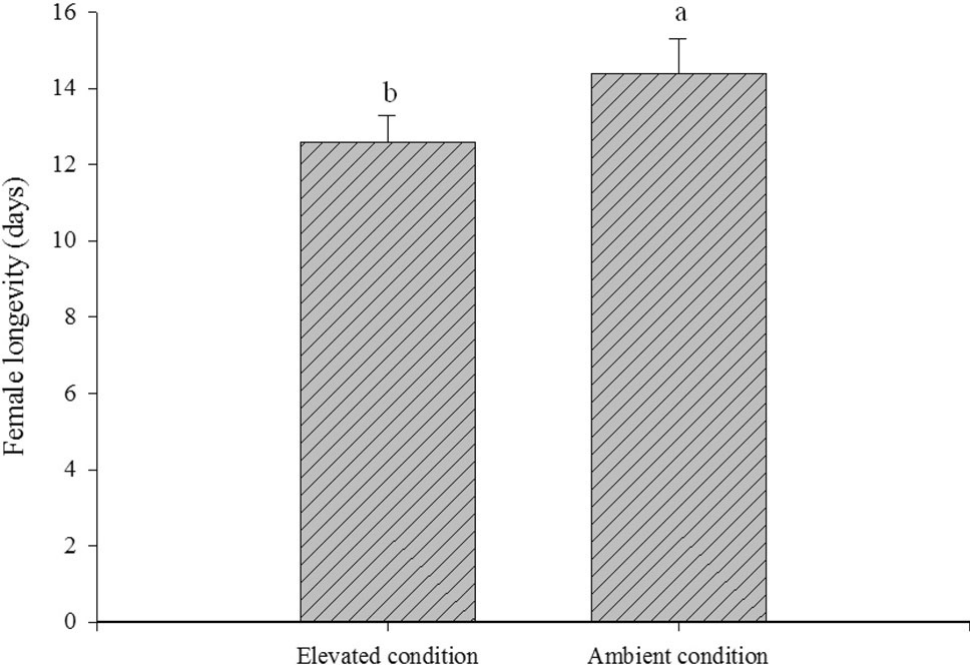
Effect of elevated CO_2_ and temperature on brown planthopper female longevity (mean ± SE)

**Fig. 7:**
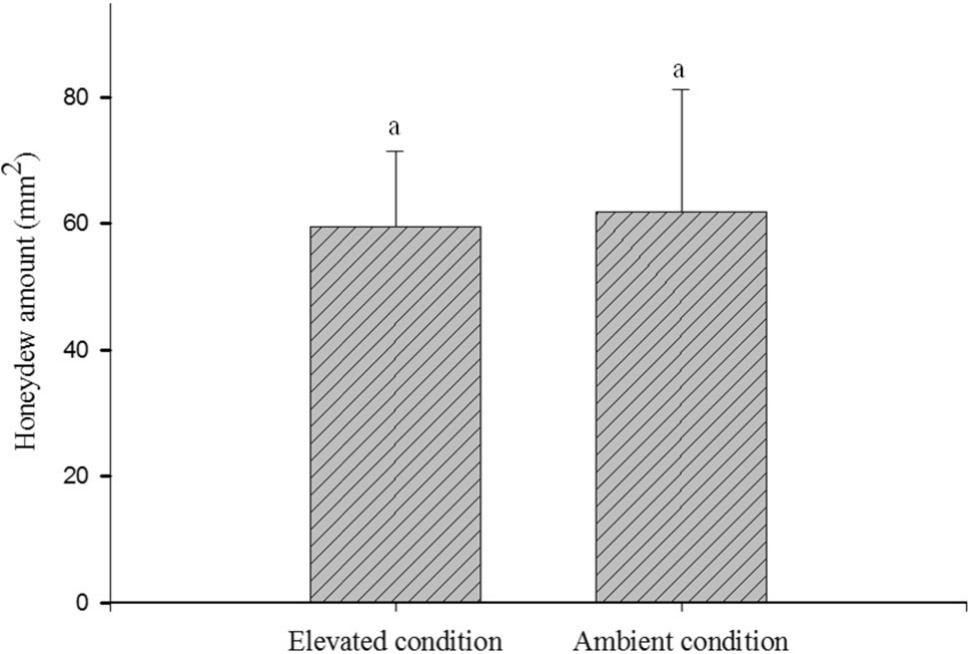
Effect of elevated CO_2_ and temperature on brown planthopper honeydew excretion (mean ± SE)

Further, developmental period of nymphs (t = 3.9, *P* = 0.001) and longevity of brachypterous females (t = 2.3, *P* < 0.05) were significantly reduced under elevated condition as compared to ambient. Elevated treatment thus reduced the life span by 18.5 and 17.4 % for nymphs (Fig. 5) and females (Fig. 6) respectively. The amount of honeydew excreted by the brachypterous female did not significantly differ under elevated and ambient condition which ranged from 59.6 ± 11.9 and 61.8 ± 19.5 mm^2^ respectively (Fig. 7).

**Fig. 8:**
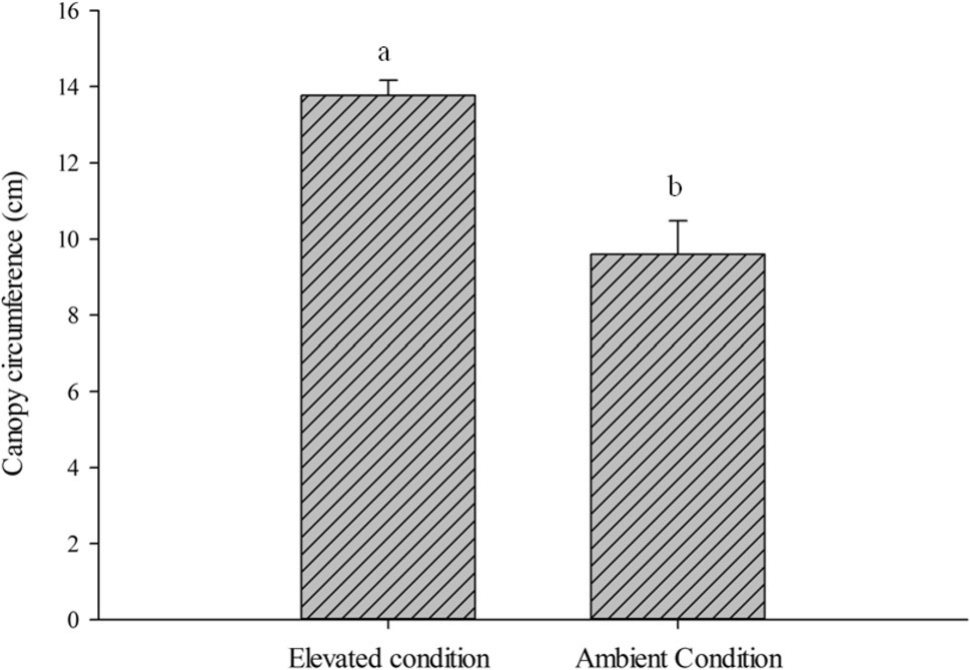
Effect of elevated CO_2_ and temperature on canopy circumferences (at 50–55 DAT) of Pusa Basmati 1401 (mean ± SE)

In rice crop, interactive effect of elevated CO_2_ and temperature led to significant increase in the number of tillers (t = 2.2, *P* = 0.04 %) and canopy circumference (t = 4.3, *P* = 0.0003 %, Fig. 8). However, grain yield was reduced under elevated condition (30.4 ± 2.2 g) as compare to ambient conditions (33.7 ± 1.2 g). This could be attributed to decrease in number of reproductive tillers (10.8 %), seeds/panicle (10.9 %) and 1000 seed weight (8.6 %) under elevated conditions in contrast to ambient. Irrespective of the nutritive effect of elevated CO_2_ on rice crop, higher yield loss of 30.6 % was observed under elevated conditions than ambient 22.3 % due to increased BPH population (Table 2).

**Table 2:** Rice growth and yield parameters under elevated (CO_2_ + temperature) and ambient (CO_2_ + temperature) condition

Parameters*	Uninfested			Infested		
	Ambient condition	Elevated condition	‘t’ statistics	Ambient condition	Elevated condition	‘t’ statistics
No. of tillers/hill	21.9 ± 1.8	27.4 ± 1.7	t = 2.2 (*P* = 0.04)	23.1 ± 1.2	18.4 ± 1.7	t = 1.5^NS^
No. reproductive tillers/hill	20.3 ± 2.1	18.1 ± 1.6	t = 0.8^NS^	18.4 ± 1.2	14.9 ± 1.1	t = 1.7^NS^
Seeds/panicles	88.5 ± 7.4	78.9 ± 9.0	t = 0.8^NS^	81.2 ± 8.2	73.7 ± 6.8	t = 0.7^NS^
1000 seed weight (g)	20.9 ± 0.6	19.1 ± 1.2	t = 1.3^NS^	17.2 ± 1.1	16.9 ± 1.5	t = 0.4^NS^
Yield (g)	33.7 ± 1.2	30.4 ± 2.0	t = 1.7^NS^	26.2 ± 2.6	21.1 ± 3.1	t = 1.3^NS^
Yield loss (%)	–	–		22.3	30.6	

*NS* non-significant

* Average of ten replications

Due to shorter life span, high reproductive potential and physiological sensitivity to temperature, insects are more readily amenable to climate change. The climatic change would thus have vital impact on the distribution pattern and abundance of insects. Results of this study are consistent with some earlier reports, wherein, BPH *N. lugens* [21, 26] wheat aphid, *Sitobion avenae* [27] and potato aphid, *Macrosiphum euphorbiae* [28] populations increased under elevated CO_2_ in comparison with ambient CO_2_. The increase in the BPH population could mainly be attributed to its increased fecundity and increased number of brachypterous females that might be probably due to more congenial micro-climate under dense canopy induced by elevated CO_2_. Soybean aphid, *Aphis glycines* populations under elevated CO_2_ were significantly greater after first week and attained twice the size as compared to ambient CO_2_ [29]. Combined effects of both elevated temperature and CO_2_ altered the plant phenology and pest biology and aggravated the damage by corn leaf aphid, *Rhopalosiphum maidis* and potato aphid, *M. euphorbiae* on their host plants [30, 31].

In the present study under elevated condition the developmental period of BPH nymphs and longevity of brachypterous females were found to be reduced. It has been demonstrated earlier that the combination of elevated CO_2_ plus temperature significantly reduced the nymphal and adult developmental period of corn leaf aphid, *R. maidis* [30] and yellow sugarcane aphid, *Sipha flava* [32]. Though elevated CO_2_ affects the insects indirectly but temperature acts as dominant factor as it affects the development duration directly, so under the high temperature, below the species threshold limit, insect response with increased rate of development that results in less time between generations. It has been observed earlier that every degree rise in global temperature, the life cycle of insect would be shorter. The quicker the life cycle, the higher will be the population of pests [30–32]. Higher fecundity of BPH in the present study ultimately resulted in proliferated BPH population under elevated condition than ambient condition. This has been reported earlier in case of cotton aphid, *Aphis gossypii* [33]; grain aphid, *S. avenae* [27] and peach aphid, *Myzus persicae* [34]; brown planthopper *N. lugens* [21] and corn leaf aphid *R. maidis* [30] while, decrease in fecundity was observed in case of woolly beech aphid, *Phyllaphis fagi* [35] red spider mite, *Tetranychus urticae* [36] and pea aphid, *Acyrthosiphon pisum* [37].

Quantification of honeydew was directly related to the sucking rate. The present study revealed that honeydew excretion under elevated condition did not differ significantly from ambient condition. Earlier increase in temperature alone was found to negatively affect BPH feeding rate [38, 39], while there was no significant difference in sucking under interactive effect of elevated CO_2_ and temperature [39].

Elevated condition increased 20.1 % tillers in present study which eventually improved the plant density and growth which could be manifested as increase in canopy size by 30.4 % which provides a congenial micro-environment for BPH multiplication. Previous studies revealed that plants exposed under elevated CO_2_ showed enhanced photosynthetic rate and lower respiration accredited for doubling of the tillers [40–42]. Likewise increased temperature also increased number of tillers during vegetative period [42, 43].

In the present study despite increase in number of tillers and canopy circumference in uninfested plants, grain yield was found to be reduced by 9.8 % under elevated condition than ambient. Earlier it has been found that interaction of elevated temperature and CO_2_ significantly affected seed number and yield in rice hybrids [13, 44] and rape seed [45]. Prior studies have also shown that adverse effect of high temperature was partially ameliorated by increased concentration of CO_2_ [42, 46]. It has been previously observed that grain yield increased by 40 % at high CO_2_ condition due to extra carbohydrate production at temperatures which do not cause sterility [21, 47]. Inspite of the nutritive effect of CO_2_, elevated temperatures negatively affect crop growth and yield regardless of CO_2_ concentration [48]. Thus grain yield was affected much more strongly by temperature than CO_2_ treatment.

Current study revealed that BPH caused more yield loss under elevated condition than ambient which was manifested as severe hopper burns. Earlier reports also suggested that rice crop suffered by hopper burn under elevated CO_2_ than ambient which resulted more yield loss [21]. Rising concentration of CO_2_ will improve plant growth but at the same time it may also raise the damage level by some phytophagus insects [49]. Temperature being a prime factor directly affects the insect development and survival while, elevated CO_2_ indirectly affects them via. certain plant nutrients, such as nitrogen content that are related to insect reproduction. For all the insect species, higher temperatures, below the species upper threshold limit, will result in faster development and rapid increase in pest population as the time to reproductive maturity will be reduced considerably. Hence, combined effect of elevated CO_2_ and temperature might aggravate pest damage to plants.

## Conclusion

Increased CO_2_ and temperature resulted in escalated BPH multiplication through increase in both fecundity and number of adults, thus inflicting higher yield loss in rice under elevated condition. It can thus be concluded that BPH population significantly increases under interactive influence of elevated CO_2_ and higher temperature thereby increasing yield loss. However, there is a need to gather more information on both abiotic and biotic factors at multitrophic levels to predict the impact of changing climate on insect population dynamics and crop-pest interactions in the future.
